# Clinical application of CT-assisted body surface localization combined with intraoperative stereotactic anatomical localization in thoracoscopic lung nodule resection: a single-centre retrospective study

**DOI:** 10.1186/s13019-024-02923-4

**Published:** 2024-06-28

**Authors:** Xiao Zhu, Zhi Chen, Kun-Lun Zhu, Shao Zhou, Fu-Bao Xing, Wen-Bang Chen, Lei Zhang

**Affiliations:** 1 Department of Thoracic Surgery, The First Affiliated Hospital of Bengbu Medical University, Bengbu, 233000 Anhui Province China; 2 Bengbu Medical University, Bengbu, 233000 Anhui Province China; 3 Department of Thoracic Surgery, Nanfang Hospital Southern Medical University, Guangzhou, 510000 Guangdong Province China

**Keywords:** Lung nodule, CT-guided, Localization methods, Microcoil, Thoracoscopic surgery

## Abstract

**Background:**

Today, the detection rate of lung nodules is increasing. Some of these nodules may become malignant. Thus, timely resection of potentially malignant nodules is essential. However, Identifying the location of nonsurface or soft-textured nodules during surgery is challenging. Various localization techniques have been developed to accurately identify lung nodules. Common methods include preoperative CT-guided percutaneous placement of hook wires and microcoils. Nonetheless, these procedures may cause complications such as pneumothorax and haemothorax. Other methods regarding localization of pulmonary nodules have their own drawbacks. We conducted a clinical study which was retrospective to identify a safe, accurate and suitable method for determining lung nodule localization. To evaluate the clinical value of CT-assisted body surface localization combined with intraoperative stereotactic anatomical localization in thoracoscopic lung nodule resection.

**Methods:**

We retrospectively collected the clinical data of 120 patients who underwent lung nodule localization and resection surgery at the Department of Thoracic Surgery, First Affiliated Hospital of Bengbu Medical College, from January 2020 to January 2022. Among them, 30 patients underwent CT-assisted body surface localization combined with intraoperative stereotactic anatomical localization, 30 patients underwent only CT-assisted body surface localization, 30 patients underwent only intraoperative stereotactic anatomical localization, and 30 patients underwent CT-guided percutaneous microcoil localization. The success rates, complication rates, and localization times of the four lung nodule localization methods were statistically analysed.

**Results:**

The success rates of CT-assisted body surface localization combined with intraoperative stereotactic anatomical localization and CT-guided percutaneous microcoil localization were both 96.7%, which were significantly higher than the 70.0% success rate in the CT-assisted body surface localization group (*P* < 0.05). The complication rate in the combined group was 0%, which was significantly lower than the 60% in the microcoil localization group (*P* < 0.05). The localization time for the combined group was 17.73 ± 2.52 min, which was significantly less than that (27.27 ± 7.61 min) for the microcoil localization group (*P* < 0.05).

**Conclusions:**

CT-assisted body surface localization combined with intraoperative stereotactic anatomical localization is a safe, painless, accurate, and reliable method for lung nodule localization.

## Background

With the widespread application of chest CT and increased attention given to lung cancer screening during health check-ups, the detection rate of lung nodules is increasing [1–3]. Some of these nodules may become malignant [4]. Recent statistics indicate that lung cancer is the leading cause of cancer-related deaths [5]. Thus, timely resection of potentially malignant nodules is essential. Several studies suggest that for adenocarcinoma in situ (AIS) and minimally invasive adenocarcinoma (MIA), the prognosis of sublobar resection is statistically similar to that of lobectomy [6, 7]. On the other hand, video-assisted thoracic surgery (VATS) is both feasible and safe, and its long-term efficacy is comparable to that of open thoracic surgery in terms of reduced patient pain and surgical complications relative to open thoracic surgery [8]. Consequently, sublobar resection via thoracoscopy is the first choice for surgeons treating small lung nodules.

However, thoracoscopic sublobar resection poses challenges for surgeons [9]. They cannot directly palpate the nodule, nor can they hastily resect the lobe. Identifying the location of non surface or soft-textured nodules during surgery is challenging. Various localization techniques have been developed to accurately identify lung nodules [9–11]. Common methods include preoperative CT-guided percutaneous placement of hook wires and microcoils. Nonetheless, these procedures may cause complications such as pneumothorax and haemothorax and increase the risk of displacement [12–16]. Other methods involve CT-guided percutaneous placement of radioactive particles or dyes such as methylene blue, but these also lead to complications and additional challenges [17]. The placement of radioactive particles for lung nodule localization exposes both medical staff and patients to additional radiation. Injection of methylene blue for localization can result in dye diffusion, affecting the accurate identification of the site if surgery is not promptly performed [18]. Preoperative electromagnetic navigational bronchoscopy (ENB) is safe and precise for injecting indocyanine green or iodized oil [19–22] but is complex, costly, and difficult to generalize. Intraoperative ultrasound is a non invasive and straightforward approach [23, 24] but may fail if the lung tissue is poorly collapsed or contains gas. Three-dimensional reconstruction is non invasive but requires additional time and technical expertise to create a lung model [25]. 3D printing has been proven to be effective [26] but is not widely accessible due to the lack of necessary equipment in many hospitals. Several researchers [27, 28] have developed intricate methods using virtual lung mapping techniques combined with intraoperative fluoroscopic navigation, but these techniques have not yet been widely adopted. Intraoperative near-infrared fluorescence imaging is safe and effective [29, 30] but is limited by equipment requirements and penetration depth and may be affected by inflammation.

To identify a safe, accurate and suitable method for determining lung nodule localization, a retrospective analysis was conducted on the clinical data of 120 patients who underwent lung nodule localization and resection surgery at the Department of Thoracic Surgery, First Affiliated Hospital of Bengbu Medical College, between January 2020 and January 2022. This analysis compared Group A, consisting of 30 patients who underwent CT-assisted body surface localization combined with intraoperative stereotactic anatomical localization, with Groups B, C, and D, each comprising 30 patients. The groups were subjected to different methods of localization.

## Methods

We retrospectively collected the clinical data of 120 patients who underwent lung nodule localization and resection surgery at the Department of Thoracic Surgery, First Affiliated Hospital of Bengbu Medical College, from January 2020 to January 2022. These patients were randomly selected according to our inclusion criteria for this study. Among them, 30 patients underwent CT-assisted body surface localization combined with intraoperative stereotactic anatomical localization, 30 patients underwent only CT-assisted body surface localization, 30 patients underwent only intraoperative stereotactic anatomical localization, and 30 patients underwent CT-guided percutaneous microcoil localization. All clinical information was from the hospital medical record. The success rates (The success rate of localization was defined by the rate of successful one-time localization, where a distance ≤ 1.5 cm between the nodule and the marking was considered successful, while a distance > 1.5 cm was considered failed localization. Regardless of the method of lung nodule localization, a localization failure is still recognized during surgery if the lung nodule is not found in the lung tissue that has been cut down, and then the operator re-searches for the nodule, and eventually the nodule is found in the lung tissue that has been cut down a second time.), complication rates, and localization times (CT-Assisted Body Surface Localization (Method for Group B) 's time consists of two parts, the first part is started when the patient is positioned on the CT examination table and stopped when the puncture point is drawn on the body surface. The second part is on the operating table when the operator holds the puncture needle, and stops the timing at the end of the puncture. Intraoperative Stereotactic Anatomical Location (Method for Group C)'s time: start the timer when the surgeon observes the patient's thoracic cavity during surgery, and stop the timer when the surgeon leaves a cautery mark on the lung surface with the electrocoagulation knife. CT-Assisted Body Surface Localization Combined with Intraoperative Stereotactic Anatomical Localization (Method for Group A)'s time is equal to CT-Assisted Body Surface Localization (Method for Group B)'s time plus Intraoperative Stereotactic Anatomical Location (Method for Group C)'s time. Time used for Preoperative CT-Guided Percutaneous Insertion of a Microcoil for Localization (Method for Group D): start the timer when the patient is positioned on the CT examination bed and stop the timer when the micro-spring coil has been placed and the puncture site is covered with gauze on the body surface). of the four lung nodule localization methods were statistically analysed.

### Inclusion and exclusion criteria

The inclusion criteria were as follows: 1. The maximum diameter of the pulmonary nodule was ≤ 30 mm. 2. The nodule was located in the outer 1/3 region of the lung field. 3. Patient age was ≤ 70 years. 4. The same pulmonary nodule localization method was used by the same person. 5. Prior to surgery the patient had already undergone a chest CT at our hospital. 6. The patient had not undergone a lung puncture biopsy or a thoracentesis.

The exclusion criteria were as follows: 1. A pleural indentation sign was present at the nodule site. 2. The solid component of the nodule was ≥ 50%. 3. Patients exhibited extensive pleural adhesion. 4. Presence of emphysema in the patient. 5. Patients had a history of radiotherapy or chemotherapy. 6. Surgical excision yielded ≥ 2 lesions. 7. Patients who underwent direct lobectomy. 8. Patients with enlarged hilar or mediastinal lymph nodes. 9. The patient had no pleural effusion.

### Technical methods

#### CT-Assisted body surface localization combined with intraoperative stereotactic anatomical localization (Method for group A)

Given the complexity of intraoperative localization and the potential possibility for anatomical markers to move during surgery, a two-pronged approach was employed. Initially, the approximate region of the nodule was determined using CT-assisted body surface localization. This approach reduces the scope of intraoperative anatomical localization, decreasing the difficulty of the process. Subsequent anatomical localization was more precise. When discrepancies arise between CT-assisted body surface localization and intraoperative stereotactic anatomical localization, two scenarios should be considered: If the patient is an elderly individual with loose skin or a female with the surface marker of the nodule near the breast area, the results of the intraoperative localization should be prioritized. Conversely, if the development of the lung fissure is suboptimal, the findings from CT-assisted body surface localization should be given precedence.

#### Preoperative CT-Assisted body surface localization (Method for group B)

**Fig. 1:**
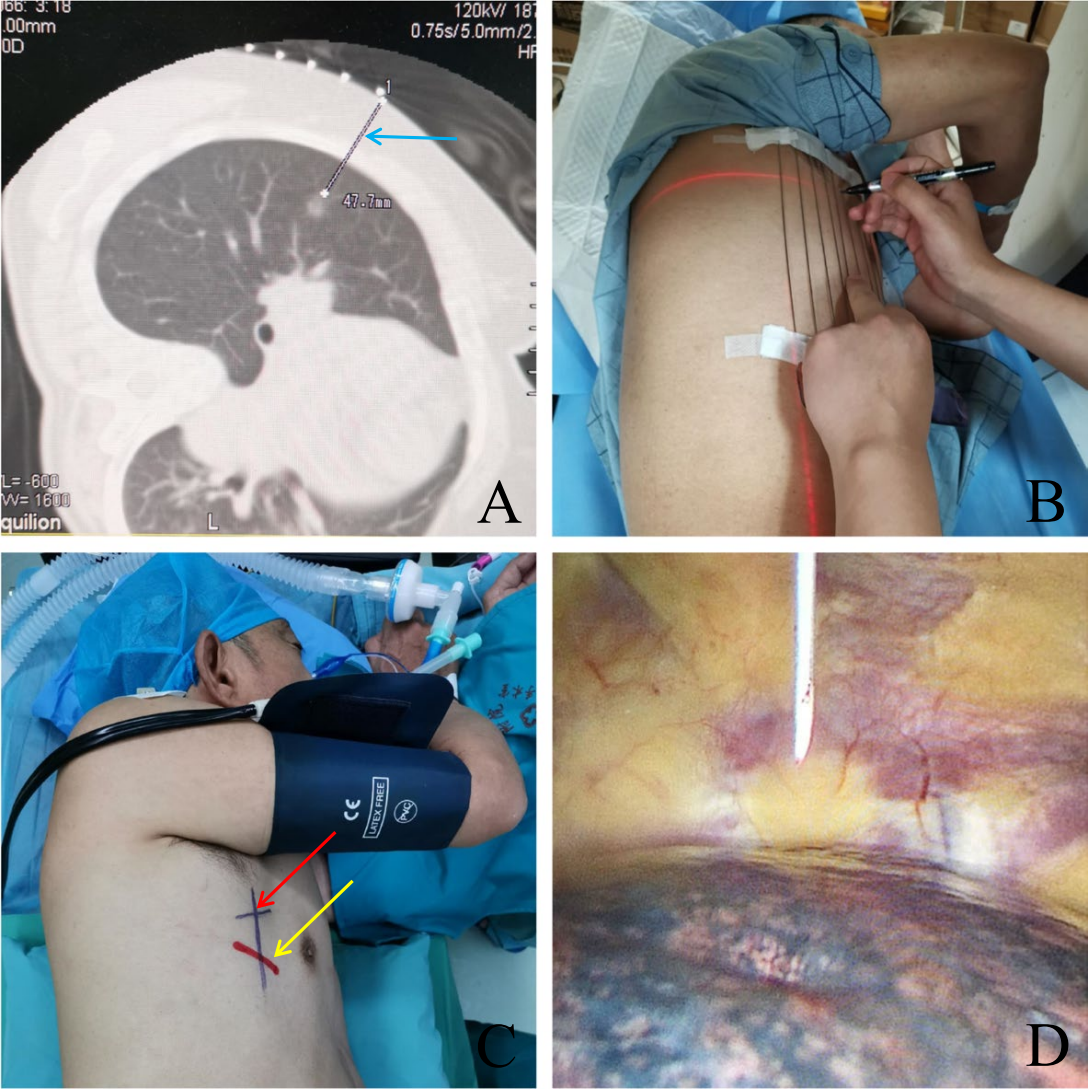
Preoperative CT-Assisted Body Surface Localization (Method for Group B). Panel **a** shows the patient's chest CT scan at the level of the nodule, with the blue arrow indicating the distance from the nodule centre to the surface. Panel **b** shows the results obtained by the clinician marking the lung nodule's projection on the skin with the assistance of the CT infrared line and metal bars. Panel **c** shows the patient after general anaesthesia and intubation in the same position as during the CT scan; the red arrow points to the lung nodule's puncture location, and the yellow arrow points to the surgical incision site. Panel **d** illustrates the insertion of the locating puncture needle into the thoracic cavity

Patients were initially positioned in the same posture as was used during the surgical procedure. A localization assistant device composed of evenly arranged metal bars was placed on the patient's chest. After the patient took a deep breath and held it, a CT scan was performed. Upon identifying the most visible level of the lung nodule, the CT machine was retracted to that level. A line, termed the "X-line", was drawn on the patient's skin along the infrared line projected by the CT machine. Another line, parallel to the body's longitudinal axis and termed the "Y-line," was drawn along the metal bar corresponding to the surface projection point of the lung nodule. The intersection of the X and Y lines marks the surface projection point of the lung nodule. The distance from this point to the nodule and the thickness of the chest wall were measured along a line termed the "Z-line". After successful induction of general anaesthesia, the patient was repositioned as during the CT scan. A puncture needle was inserted vertically into the skin at the marked point, reaching a depth 1 cm greater than the chest wall thickness measured preoperatively on the CT scan to ensure penetration into the lung tissue. The anaesthetist then inflated the patient's lung before withdrawing the puncture needle. Following localization, a chest support pad was placed beneath the patient. Video-assisted thoracic surgery was initiated. During the procedure, haemorrhagic spots left by the puncture needle on the lung surface, indicating the location of the nodule, were visualized and marked using an electrosurgical knife. The operation procedure is shown in Fig. 1.

#### Intraoperative stereotactic anatomical location (Method for group C)

**Fig. 2:**
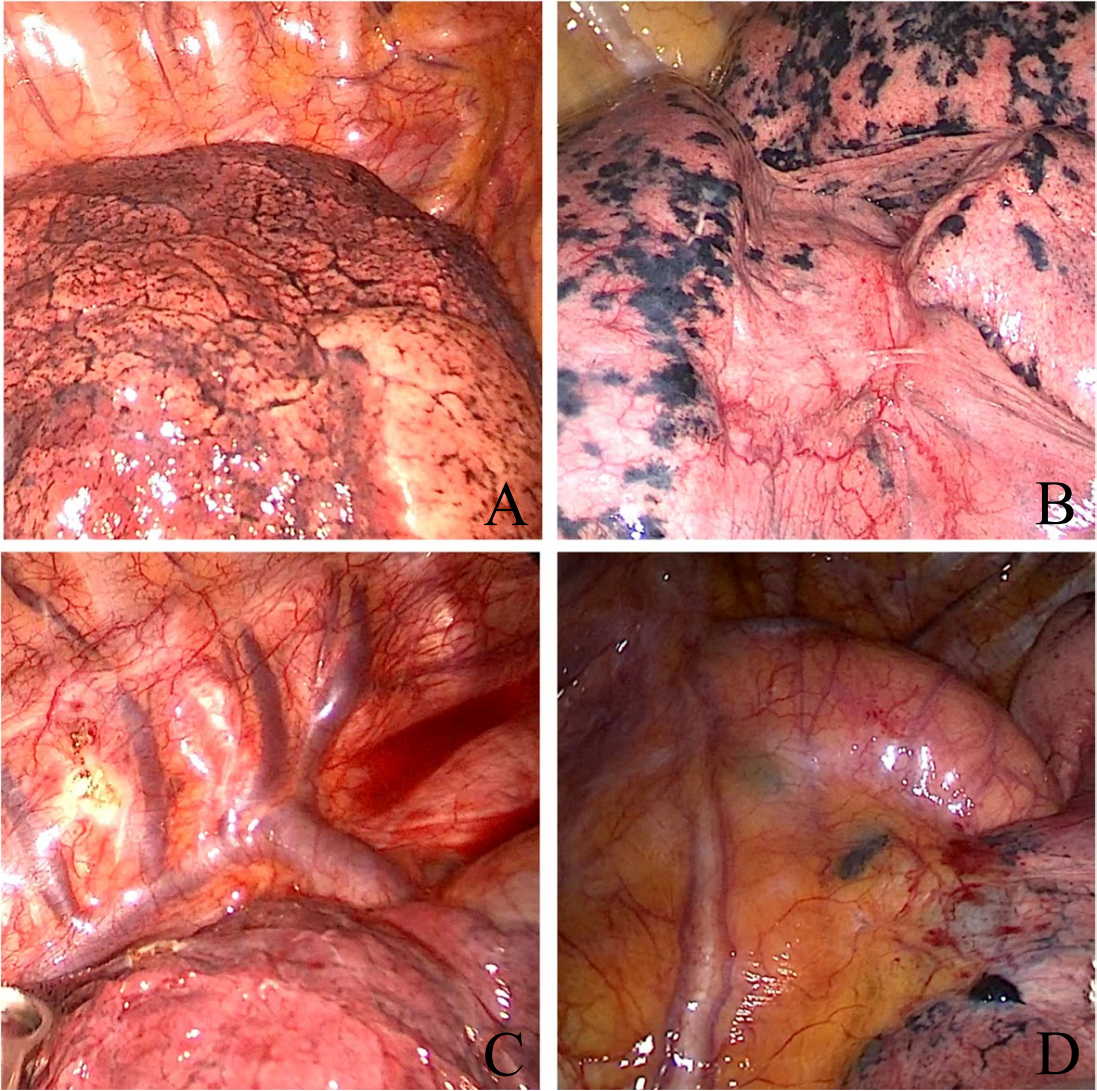
Intraoperative Stereotactic Anatomical Location (Method for Group C). The figure displays common reference markers for intraoperative anatomical localization. Panel **a** depicts the lung apex; Panel **b** displays the lung fissures; Panel **c** shows the azygos vein arch; Panel **d** represents the aorta

Upon successful induction of anaesthesia, the patient was positioned in the lateral decubitus position, and a chest support pad was placed beneath the patient. Video-assisted thoracic surgery was commenced. During surgery, inherent anatomical landmarks of the thorax, such as the lung apex, lung margin, lung fissures, aortic arch, pulmonary artery, pulmonary vein, azygos vein arch, superior vena cava, oesophageal-tracheal groove, azygos vein notch and paravertebral line, were sought. Preoperatively, through meticulous examination of the patient's thin-slice chest CT images, the relationship between the nodule and related anatomical landmarks was determined. The surgeon compared intraoperatively identified landmarks with those from preoperative CT images to localize the nodule. Notably, due to deformations between the expanded and collapsed states of the lung, proportional relationships between landmarks were consulted. The identified location was then marked using an electrosurgical knife. The operation procedure is shown in Fig. 2.

#### Preoperative CT-Guided percutaneous insertion of a microcoil for localization (Method for group D)

**Fig. 3:**
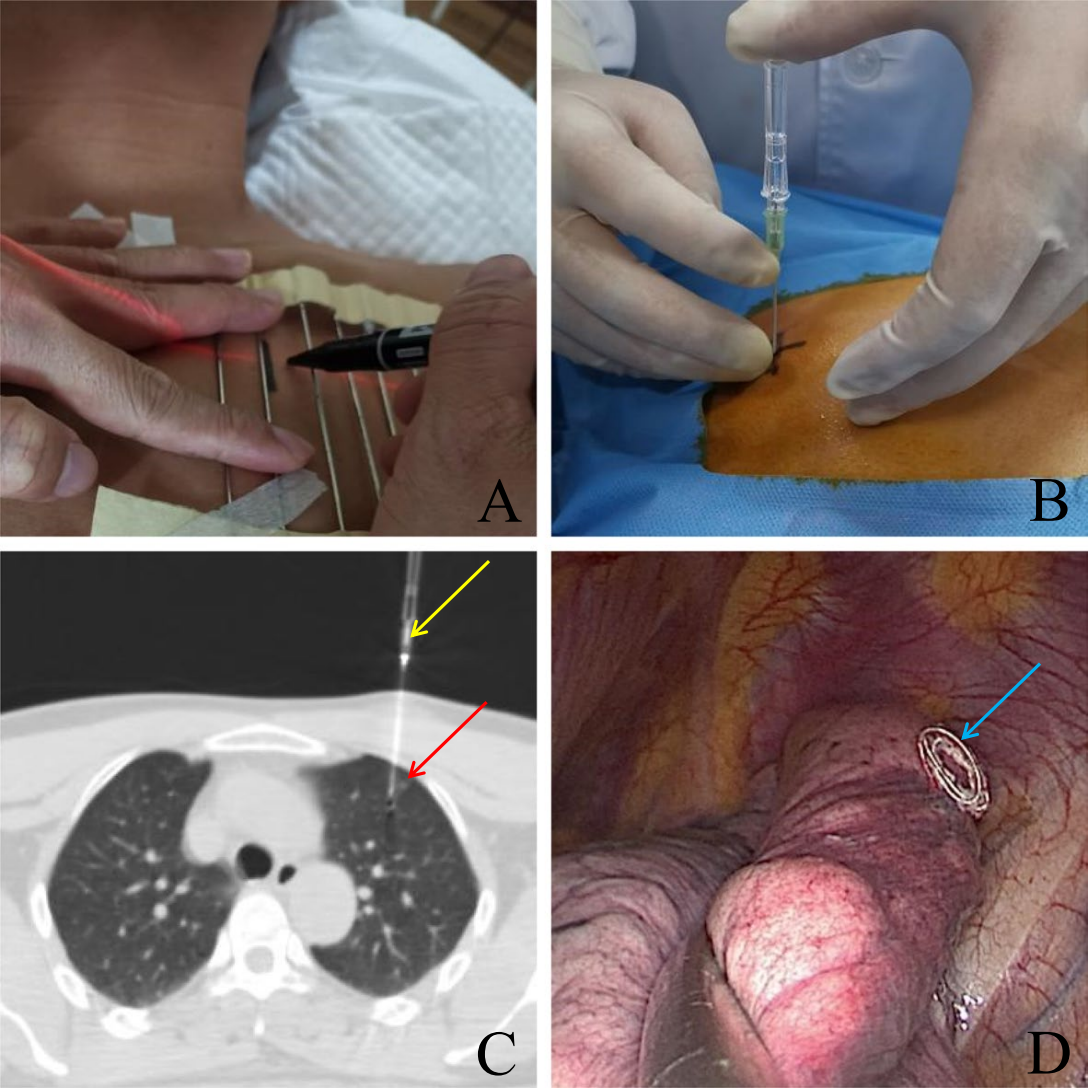
Preoperative CT-Guided Percutaneous Insertion of a Microcoil for Localization (Method for Group D). Panel **a** displays the lung nodule's projection on the skin under CT guidance. Panel **b** illustrates how the clinician inserted the puncture needle before deploying the microcoil. Panel **c** shows the CT image taken during the localization process, with the yellow arrow pointing to the puncture needle and the red arrow to the lung nodule. Panel **d** provides an intraoperative image showing the deployment of the microcoil on the lung surface

After the localization assistance device, composed of adjacent evenly arranged metal bars, was placed on the patient's chest, a chest CT scan was performed. The puncture point was determined and marked on the skin, and the depth and angle of needle insertion were established. Following aseptic preparation and local anaesthesia, the patient was instructed to hold their breath and the puncture needle was inserted into the subpleural space of the lung. This procedure was followed by another CT scan, adjustments to the needle tip position, and further CT confirmation. Once the location was identified, the microcoil was deployed, anchoring near the nodule. After the placement, another CT scan was conducted to ensure proper coil localization. The patient was monitored for potential complications, such as haemorrhage and pneumothorax. The operation procedure is shown in Fig. 3.

#### Procedure for lung nodule resection

The area of lung tissue containing the marker point of the pulmonary nodule was gently elevated by the surgeon using atraumatic forceps. Subsequently, to ensure an adequate surgical margin, wedge resection of the lung tissue encompassing the nodule was performed using an endoscopic linear cutter. When the lung tissue was resected, we used a lancet to dissect the lung tissue in a direction perpendicular to the pleural surface right up to the lung nodule, and the distance between the dissection and the locator marking point on the pleura was measured with a straightedge and recorded in the operative record. If the nodule was not identified in the initial excised lung tissue, the resection range was expanded. The resected lung tissue was subjected to rapid frozen section pathology. When rapid frozen section pathology revealed that the nodule was malignant, we performed a lobectomy. For adenocarcinoma in situ or minimally invasive adenocarcinoma, the surgical margins have been more than 2 cm.

### Statistical analysis

Correlations were analysed using SPSS 23.0 software. Quantitative data are presented as the mean ± standard deviation and One-way ANOVA was applied for analysis among different groups with Turkey post hoc test. Numerical data are presented as frequencies and percentages and were compared using chi-square tests. The level of significance was set at *P* < 0.05.

## Results

Patient information for each group (including sex, age, pulmonary lobe where the nodule was located, nature of the nodule, diameter of the nodule, and the distance from the pulmonary nodule to the visceral pleura) is presented in Table 1.

**Table 1:** Basic patient information

	Group A (*n* = 30)	Group B (*n* = 30)	Group C (*n* = 30)	Group D (*n* = 30)	χ^2^/*F*	*P*
Sex					0.804	0.849
Male	13 (43.3)	13 (43.3)	14 (46.7)	16 (53.3)		
Female	17 (56.7)	17 (43.3)	16 (53.3)	14 (46.7)		
Age (in years)	48.80 ± 11.17	54.47 ± 10.94	51.77 ± 9.20	47.03 ± 13.16	2.567	0.058
Location of Nodule						
LUL	9 (30.0)	8 (26.7)	9 (30.0)	6 (20.1)		
LLL	7 (23.3)	6 (20.0)	7 (23.3)	7 (23.3)		
RUL	7 (23.3)	7 (23.3)	7 (23.3)	10 (33.3)		
RML	1 (3.3)	1 (3.3)	2 (6.7)	1 (3.3)		
RLL	6 (20.1)	8 (26.7)	5 (16.7)	6 (20.1)	0.268	0.966
Nature of Nodules						
PSN	13 (43.3)	14 (46.7)	15 (50.0)	14 (46.7)		
GGN	17 (56.7)	16 (53.3)	15 (50.0)	16 (53.3)	2.567	0.058
Diameter (mm)	11.90 ± 4.40	12.03 ± 3.36	13.17 ± 4.11	11.83 ± 3.28	0.811	0.490
Distance from pulmonary nodule to pleura (mm)	12.97 ± 5.78	13.07 ± 4.52	14.40 ± 5.83	14.73 ± 5.01	0.873	0.457

*Abbreviations: LUL* left upper lobe, *LLL* left lower lobe, *RUL* right upper lobe, *RML* right middle lobe, *RLL* right lower lobe, *PSN* part-solid nodule, *GGN* ground glass nodule

### Success rate of localization

Groups A and D exhibited the highest success rates, with 29 successful localizations and one failure each, achieving a 96.7% success rate. Group B had 21 successful procedures and nine failures, a 70% success rate, and Group C achieved an 86.7% success rate, with 26 successes and four failures. The success rate in Groups A and D were significantly higher than that in Group B (*P* < 0.05).

### Complication rate of localization

Groups A, B, and C all had a 0% complication rate, as the marking step was performed during surgery. Group D experienced 18 complications, including seven cases of pneumothorax, four cases of haemothorax, and seven cases of pain (It was scored according to Wong-Baker faces pain scale revision, (FPS-R). Pain was considered a complication when it reached a score of 6 (moderate pain)), for a total complication rate of 60%. The complication rate in Groups A, B, and C were significantly lower than that in Group D (*P* < 0.05).

### Comparison of localization times

The average localization time for Group A was 17.73 ± 2.52 min that for Group B was 12.57 ± 2.56 min, that for Group C was 14.37 ± 4.63 min, and that for Group D was 27.27 ± 7.61 min. The localization time in Group A was longer than those in Groups B and C (*P* < 0.05). The localization times was the longest in the Group D in comparison to other groups (*P* < 0.05).

### Pathological results of pulmonary nodules

No significant differences were observed in the pathological results of pulmonary nodules among different groups (*P* > 0.05).

The above information is presented in Table 2.

**Table 2:** Pathology and localization results related to pulmonary nodules

	Group A (*n* = 30)	Group B (*n* = 30)	Group C (*n* = 30)	Group D (*n* = 30)	F	*P*
Final diagnoses					0.229	0.876
Benign	3 (10.0)	3 (10.0)	2 (6.7)	2 (6.7)		
Atypical adenomatous hyperplasia	1 (3.3)	1 (3.3)	1 (3.3)	2 (6.7)		
AIS	15 (50.0)	13 (43.3)	14 (46.7)	12 (40.0)		
MIA	8 (26.7)	8 (26.7)	9 (30.0)	9 (30.0)		
Invasive adenocarcinoma	3 (10.0)	5 (16.7)	3 (10.0)	5 (16.7)		
Squamous cells carcinoma	0 (0.0)	0 (0.0)	1 (3.3)	0 (0.0)		
Successful Localization					4.709	0.004
Yes	29 (96.7)	21 (70.0)^a^	26 (86.7)	29 (96.7)^b^		
No	1(3.3)	9(30.0)	4(13.3)	1(3.3)		
Localization complications					43.500	0.000
No	30 (100.0)	30 (100.0)	30 (100.0)	12 (40.0)^a,b,c^		
Yes	0 (0.0)	0 (0.0)	0 (0.0)	18 (60.0)		
Localization time (min)	17.73 ± 2.52	12.57 ± 2.56^a^	14.37 ± 4.63^a^	27.27 ± 7.61^a,b,c^	55.855	0.000

^a^Represents a significant difference compared to Group A

^b^Represents a significant difference compared to Group B

^c^Represents a significant difference compared to Group C

## Discussion

Currently, an increasing number of pulmonary nodules are being detected [3], and timely removal of potentially malignant nodules is crucial. Without direct lung lobectomy, the key to surgery is how to accurately locate pulmonary nodules. Various localization techniques, such as CT-guided percutaneous hook-wire and microcoil localization, body surface theodolitic puncture localization [31] and intraoperative stereotactic anatomical localization, are applied in VATS. However, each method has its limitations. This study compared several methods, including CT-assisted body surface localization combined with intraoperative stereotactic anatomical localization, to analyse the clinical application value of these localization methods.

CT-assisted body surface localization combined with intraoperative stereotactic anatomical localization is a novel method comprising preoperative CT-assisted body surface localization and intraoperative anatomical localization. Preoperative CT-assisted body surface localization is innovative in that it is derived from conventional surface localization [31] and determines the surface projection point of the pulmonary nodule during the CT scan. This method is inherently more accurate than using anatomical structures (such as the clavicle midline and anterior midline) for localization.

When employing CT-assisted body surface localization in practice, several considerations are crucial. First, after determining the nodule's surface projection point in the CT room, it is advisable to draw an extended line on the skin. This is because if only a small ' + ' sign is marked, skin laxity can lead to deviations in the marked point. Second, after the patient has been successfully anaesthetized and intubated in the operating room, it is imperative to ensure that the patient's position mirrors that during CT-assisted body surface marking. When inserting the puncture needle, anaesthetists should ensure the lung remains inflated, simulating the patient's breath-holding state during the CT scan and minimizing potential deviations due to lung volume changes. Third, during needle insertion, it is essential to ensure that the needle is perpendicular to the skin. This localization technique allows for only one attempt, unlike CT-guided percutaneous microcoil localization, which permits multiple angle adjustments under CT guidance. If the needle is not inserted vertically, misalignment may occur. Fourth, regarding the timing of needle insertion, Zhang et al. [32] suggests first placing the thoracoscope, inserting the needle under thoracoscopic observation, then allowing the anaesthetist to inflate the lungs, and finally marking the lung surface. Conversely, we first placed a puncture needle, expanded the lung and then marked the surface of the lung, and then placed a chest support pad before surgery. This method prevents the skin from moving, displacing the surgical incisions and causing deviations in body surface markings. In the same way, the placement of chest support pads can also cause this deviation.

The advantage of CT-assisted body surface localization for pulmonary nodules is that localization can be conducted during preoperative high-resolution CT, negating the need for multiple scans, as with CT-guided percutaneous microcoil localization. This minimizes patient exposure to excessive radiation. Another benefit of this method is that the localization puncture is performed after anaesthesia, reducing patient discomfort. Patients who underwent surgery immediately after localization were considered virtually free of complications. However, this technique has its limitations. In our study, 9 of the procedure groups failed to achieve accurate localization, yielding a 70% success rate. An analysis of the failed cases revealed that six were females, seven were aged 55 or older, and seven had nodules located in the lower lobes. The results are due to the following three points: first, the high degree of movement in the female breast area leads to the movement of body surface marks; second, the less elastic skin of elderly patients can also lead to the movement of body surface markers; and finally, localization mostly fails in cases where the nodules are located in the lower lobe because the movement of the lower lobe is greater than that of the upper lobe during breathing. In addition, CT-assisted localization of the body surface is also challenging. If the points marked on the body surface are blocked by ribs, the needle cannot be inserted vertically, thus requiring the needle to be inserted at an angle. This angle is not known, so localization is likely to fail. The area blocked by the scapula cannot be located by this method.

Intraoperative stereotactic anatomical localization is a non invasive method for pinpointing lung nodules based on anatomical landmarks, such as the arc of the vena cava, aorta, pulmonary arteries and veins, lung base, lung apex, lung edges, and lung fissures [30]. Mastery of this method is challenging. During the procedure, it is crucial to maintain the lung's natural state after collapse, avoiding influences from gauze compression or repeatedly turning the lung lobe and other actions. Furthermore, practitioners should deepen their understanding of lung anatomy, enhance the ability to read high-resolution chest CT images, and develop spatial imagination capabilities. After localization, it is advised to extend the excision margin to increase the chances of complete nodule removal. Once the lung nodule is not found during the first wedge resection, it is difficult to locate the nodule again because the normal morphology of the lung has been destroyed. In addition, before surgery, the doctor did not determine the extent of the patient's lung fissure development or whether the patient's intraoperative lung collapse was ideal, and the localization method is more dependent on the subjective experience of the operator. In contrast, CT-assisted body surface localization was used to identify the relative area of the nodule to reduce the difficulty of the intraoperative stereotactic anatomical localization process.

Our findings demonstrated that similar to CT-guided percutaneous insertion of a microcoil for localization, CT-assisted body surface localization combined with intraoperative stereotactic anatomical localization is highly successful for localization. Compared with CT-guided percutaneous insertion of a microcoil for localization, this localization method also boasts benefits such as reduced localization time, low localization complication rates, decreased radiation exposure, and cost savings for patients. Although this localization method might take longer than CT-assisted body surface localization alone, its success rate is notably higher. Furthermore, the success rate of this localization method is higher than that of standalone intraoperative stereotactic anatomical localization. Although the difference was not statistically significant, increasing clinical application might magnify this difference.

Given the small sample of data used in this study, further research is warranted to substantiate our findings. In conclusion, CT-assisted body surface localization combined with intraoperative stereotactic anatomical localization is a safe, painless, accurate and cost-effective technique that merits wider adoption.

## Conclusion

CT-assisted body surface localization combined with intraoperative stereotactic anatomical localization is a safe, painless, accurate, and reliable method for lung nodule localization.

## Data Availability

Data is provided within the manuscript or supplementary information files.

## Abbreviations

AIS: Adenocarcinoma in situ
MIA: Minimally invasive adenocarcinoma
VATS: Video-assisted thoracic surgery
LUL: Left upper lobe
LLL: Left lower lobe
RUL: Right upper lobe
RML: Right middle lobe
RLL: Right lower lobe
PSN: Part-solid nodule
GGN: Ground glass nodule
